# Mr. Farish's Case of Calculus

**Published:** 1807-01

**Authors:** 


### *To the Editors of the Medical and Physical Journal.*

GENTLEMEN,

I Request you will have the goodness to insert the following communication in your useful Journal. I am induced to publish it from the singularity of the circumstances with which the case is attended; and with a hope that it may tend to rouse a spirit of inquiry respecting the most painful disease to which human nature is incident, and which may be justly ranked as one of the opprobria of medical science.

*A remarkable Case of Calculus, transmitted in a letter from Thomas Farish, Esq. of Orange County, Virginia, to John Wharton, M. D. lately President of the Royal Physical Society of Edinburgh.*

BEFORE entering upon a detail of the Case, as communicated by my friend, it will be necessary, in few words, to describe his habit of life and temperament of body. Mr. Farish is a gentleman, who for many years past, has used a great deal of exercise, particularly on horseback. He has always resided in the pure air of the country, and has uniformly lived temperately. He is descended from podagric parents, and has for several years past been the victim of gout, notwithstanding every exertion to ward off this fashionable though tormenting malady. He is at present about sixty-one years of age, of a plethoric habit and sanguine temperament; and for a person of that age, possesses an uncommon degree of vivacity, in spite of the complicated evils with which he has been harrassed.

*Dean Street, Canterbury Square, Dec.* 13, 1806.

---

*Orange County, Virginia, Aug.* 15, 1805,

"My dear Sir,

"In several letters to your father, you express a desire to learn the history of my disease; therefore I shall, as briefly as possible, and as far as memory will admit, communicate the symptoms which, at different periods, the disease has assumed. Early in the spring of 1802, I was attacked with a violent nausea, and great efforts to discharge the contents of the stomach; oppressed with flatulency, and an excruciating pain extending to the scrotum. It was thought to be the bilious colic; I took calomel, and a great a great quantity of bile was discharged. My usual health again returned. In August or September, for of this I am not certain, while riding on horseback, I felt for the first time, and very suddenly, a sharp stinging sensation in the penis, accompanied with a strong inclination to expel the contents of the bladder, which was effectuated with much pain and difficulty. The urine was of a dark, soot-like colour, the burning sensation continued, and still continues, more especially when passing urine, which I am under the necessity of doing very frequently. Blood has in two instances passed off from the urethra, of a grumous consistence.

"In March, 1803, for the space of twelve hours, I suffered more pain than I ever had before or since that period. I found it utterly impracticable to void any urine; the catheter was employed, and a large quantity of sooty-coloured urine was discharged. I felt ease for eight days, when a second stoppage took place; the catheter was again had recourse to, and the urine voided had the natural appearance. Eight days afterwards a third obstruction ensued; the catheter was again used, but little water was discharged. It was employed again, in the force with which it was used; there passed a stone three-quarters of an inch long, and nearly half an inch in diameter. I felt somewhat relieved for the space of a fortnight, when the same symptoms again recurred.

"On the 23d of September, 1804, I passed by stool, with much pain, a stone of the following dimensions, one inch and seven-eights in length, and one inch and one eight in diameter. For the space of eighteen months prior to the discharge of this calculus, by pressing my hands upon my buttocks, I felt a sensation there, like that occasioned by a pricking substance. I seldom feel any thing similar at present.

"I am still much afflicted with stone. Sharp gravel, of a yellow colour, are frequently discharged. Seven or eight times this spring and summer, I have passed small pebbles of a very rugged appearance, and also of a yellow colour. I am much troubled with nausea and vomiting, combined with my old winter companion, the gout. I am just able to partake of my favourite amusement, by riding on horseback, but at a very slow pace indeed. I walk, but at a slow and solemn pace. I still keep my flesh, and my usual good flow of spirits. With every sentiment of respect, I remain yours, sincerely."

